# Kaposi sarcoma herpesvirus (KSHV)-associated lymphomas are associated with markedly elevated serum IL-10, elevated IL-6, IL-17 and circulating KSHV

**DOI:** 10.1186/1750-9378-7-S1-P39

**Published:** 2012-04-19

**Authors:** Thomas S Uldrick, Mark N Polizzotto, Kathleen Wyvill, Karen Aleman, Vickie Marshall, Richard F Little, Armando Filie, Mark Raffeld, Seth M Steinberg, Stefania Pittaluga, Denise Whitby, Robert Yarchoan

**Affiliations:** 1 HIV and AIDS Malignancy Branch, Center for Cancer Research, National Cancer Institute, National Institutes of Health, Bethesda, MD, USA; 2 Laboratory of Pathology, Center for Cancer Research, National Cancer Institute, National Institutes of Health, Bethesda, MD, USA; 3 Biostatistics and Data Management Section, Center for Cancer Research, National Cancer Institute, National Institutes of Health, Bethesda, MD, USA; 4 AIDS and Cancer Virus Program, SAIC-Frederick, National Cancer Institute, National Institutes of Health, Frederick, MD, USA

## Background

KSHV, also called human herpesvirsus-8 (HHV8), is the etiologic agent of primary effusion lymphoma (PEL) (including extracavitary variant), and large B-cell lymphoma arising in HHV8-associated multicentric Castleman disease (together, KSHV-associated non-Hodgkin lymphoma (KSHV-NHL). Additional KSHV-associated diseases include: Kaposi sarcoma (KS), a form of multicentric Castleman disease (KSHV-MCD), and a proposed KSHV-associated inflammatory cytokine syndrome (KICS). Like KSHV-MCD, inflammatory symptoms are common in KSHV-NHL. We compared an array of inflammatory and angiogenic cytokines, chemokines, growth factors, and select clinical laboratory values between HIV-infected patients with KSHV-NHL and other lymphomas (HIV-lymphoma).

## Methods

Patients were enrolled in HAMB and/or NIAID protocols. Cases had KSHV-NHL; controls other HIV-lymphoma. Clonality of PEL diagnosed from effusions was confirmed by PCR for immunoglobin rearrangements. Serum was evaluated by ELISA for IFN-γ, IL-1β, IL-6, IL-8, IL-10, IL-12p70, TNF-α, IL-17, VEGF-A, (Meso-Scale Discovery, Gathersberg, MD), CXCL1, VEGF-C (R&D Systems, Minneapolis, MN). In patients with KSHV-NHL, peripheral blood mononuclear cell associated KSHV viral load was measured. Clinical data included demographics, CD4 count, albumin, platelets, hemoglobin, and c-reactive protein (CRP). Comparison of each parameter between patients with KSHV-NHL and other HIV-lymphoma employed an exact form of the Wilcoxon rank-sum test. P-values are 2-sided, with p ≤ 0.01 considered statistically significant, and 0.01< p <0.05 considered strong trends.

## Results

Subjects:13 KSHV-NHL cases: 12 men, 1 woman. Median age 44, (IQR 39-55). 4 white, 4 Hispanic, 3 African-American, 2 African. PEL (8), extracavitary variant PEL (3), large B-cell lymphoma arising in HHV8-associated MCD (2, both with large effusions), history pathology confirmed KSHV-MCD (4). 28 HIV-associated lymphoma controls: 23 men, 5 women. Median age 38 (IQR 35-46). 17 white, 4 Hispanic, 6 African-American, 1 African. Histologies: primary central nervous system lymphoma (13), diffuse large B-cell lymphoma (DLBCL) (10), Hodgkin disease (1), Burkitt lymphoma (2), plasmablastic lymphoma (1), EBV+ large B-cell lymphoma NOS (1). KSHV-NHL subjects had elevated KSHV viral load, [median 2812 copies/10^6^ cells (IQR 186-115,789)] and CRP [median 51 mg/L (IQR 45-67)]. Compared to other HIV-lymphomas, patients with KSHV-NHL have higher CD4 counts (median CD4 133 vs. 29 cells/uL, p=0.002), hypoalbuminema (median albumin 1.9 vs. 3.5 mg/dL, p= 0.0034)), and trend towards more severe anemia, thrombocytopenia, and hyponatremia. KSHV-NHL is associated with elevated circulating KSHV, marked elevations in IL-10 (513 vs. 12.2 pg/mL, p <0.0001), elevations in IL-6 (29 vs. 4.1 pg/mL, p=0.0013), IL-17 (1.6 vs. 0.5 pg/mL, p=0.0074), and trends towards increased IFN-γ and IL-1β.

## Conclusions

Inflammatory cytokines are important n KSHV-NHL pathogenesis and symptomatology. Clinical and translational studies evaluating these abnormalities in KSHV-associated malignancies are ongoing.

